# Soybean Oligosaccharides Mitigate HFD-Induced Obesity in Mice with Changes in the Gut Mucus–Microbiota Axis

**DOI:** 10.3390/nu18081282

**Published:** 2026-04-17

**Authors:** Jingyi Zhang, Nana Zhang, Jing Chen, Jia Liu, Zhaosen Ge, Yifeng Zhou, Fengzhong Wang

**Affiliations:** 1 Hubei Key Laboratory of Biological Resources Protection and Utilization, Hubei Minzu University, Enshi 445000, China; oliviazhangzhang@163.com; 2 Institute of Food Science and Technology, Chinese Academy of Agricultural Sciences, Key Laboratory of Agro-Products Processing, Ministry of Agriculture, Beijing 100090, China; zhangnn16@163.com; 3 College of Food Science and Technology, Agricultural University of Hebei, Baoding 071001, China; 18232062545@163.com; 4 China National Research Institute of Food and Fermentation Industries, Beijing 100015, China; liujiajessica@163.com; 5 Beijing City University, Beijing 100094, China; 13051484372@163.com

**Keywords:** soybean oligosaccharides, intestinal mucus layer, gut microbiota, obesity, glycosyltransferase

## Abstract

**Background:** Intestinal barrier problems cause obesity and related health issues. We focus on treatments that fix the gut lining and change gut bacteria. Soy oligosaccharides (SOSs) are prebiotics. They change gut bacteria and help lower fats. The mechanism by which SOS affects high-fat diet (HFD)-induced obesity remains to be fully elucidated. **Objectives:** We want to see if SOS improves the mucus barrier in the gut by investigating how mucus is produced, modified and released. We hypothesise that SOS can reduce obesity and associated health problems by regulating mucus and gut bacteria. **Methods:** Accordingly, HFD-fed mice were used in this study. **Results:** The results showed that SOS alleviated HFD-induced weight gain and glucose disorders. It also enhanced the gut mucus barrier by promoting goblet cell differentiation and regulating mucus-related genes. In addition, SOS intervention was associated with increased abundance of potentially beneficial gut taxa. These bacterial changes were linked to better health measures. In conclusion, our findings demonstrate that SOS confer metabolic protection against HFD-induced obesity, at least partially, by coordinately modulating the mucus–microbiota axis. **Conclusions**: These data suggest that SOS may alleviate obesity and related disorders by improving the intestinal mucus layer and gut microbiota.

## 1. Introduction

Obesity is a leading global health concern and a major risk factor for type 2 diabetes, cardiovascular disease, and other chronic disorders [1]. The global burden of obesity and related diseases is projected to continue rising, underscoring the urgent need for safe, effective, and sustainable weight management strategies [2]. Although the current mainstream anti-obesity therapies (such as drug and surgical intervention) have certain effects, they are often accompanied by limitations such as weight rebound, gastrointestinal side effects and surgical complications [3]. The role of gut homeostasis in the onset and progression of obesity has received growing attention in recent years. Among these, the mucus–microbiota axis, as a core regulatory mechanism of intestinal barrier function, has emerged as a key entry point for research into metabolic diseases [4].

Gut barrier dysfunction, along with the subsequent translocation of harmful substances, has been linked to the development of obesity [5]. The mucus layer, which is mainly secreted and synthesized by goblet cells, is a vital component of the intestinal barrier [6]. MUC2, the core component of the mucus barrier, forms a protective gel layer that prevents the entry of harmful substances and pathogens [4,7]. HFD-induced gut dysbiosis impairs goblet cells and reduces MUC2 expression, leading to disrupted intestinal homeostasis and increased permeability [8,9,10]. Thus, preserving or restoring mucus barrier function represents a potential therapeutic target for obesity prevention and treatment.

Functional oligosaccharides, characterized by a low degree of polymerization, exhibit resistance to digestive enzymes and can pass through the gastrointestinal tract to reach the colon. There, they modulate gut microbiota composition and intestinal barrier integrity, thereby conferring health benefits. Oligosaccharides, including fructo-oligosaccharides and galacto-oligosaccharides, can be isolated from plant sources such as millet, sorghum, and legumes. SOSs, predominantly composed of galacto-oligosaccharides, represent key bioactive components of soybeans, accounting for approximately 5% of soybean dry matter [11,12]. SOSs are a type of natural prebiotics with various physiological functions. For example, they selectively promote beneficial gut bacteria (e.g., *bifidobacteria*), thereby improving gut microecology, enhancing immunity, facilitating mineral absorption, and helping regulate blood glucose and lipids [13]. Functional oligosaccharides are now extensively utilized in food, nutraceuticals, and animal feed worldwide. Notably, SOSs have been shown to hold promise for the prevention of various chronic conditions, such as cancer, atherosclerosis, and menopausal disorders [14,15]. However, the specific effects of SOS on the intestinal barrier, especially the expression of genes related to mucus synthesis, glycosylation, secretion and degradation, as well as corresponding histological changes, have not been fully elucidated.

Accordingly, we hypothesized that SOS alleviates HFD-induced metabolic dysregulation through modulation of the intestinal mucus barrier and gut microbiota. The present study explored the mucus–microbiota axis as a key mechanism mediating the anti-obesity action of SOS, laying a theoretical foundation for its application as a functional food component.

## 2. Materials and Methods

### 2.1. Materials

Invitrogen (Carlsbad, CA, USA) supplied the Trizol reagent. The GoScript reverse transcription (RT) system and Power SYBR Green PCR master mix were obtained from Promega (Madison, WI, USA). Soybean-derived SOSs (containing 11.8% verbascose, 37.0% stachyose, 11.1% raffinose, and 21.3% sucrose) were procured from Yuanye Biotechnology Co., Ltd. (Shanghai, China).

### 2.2. Structural Characterization

#### 2.2.1. Molecular Weight (Mw) Analysis

A gel column (Shodex SB-806, Showa Denko, Tokyo, Japan) coupled with a combined detector system (Wyatt Dawn Heleos II eight-angle laser scattering detector and Wyatt Optilab rEX differential refractive detector, Wyatt Technology, Santa Barbara, CA, USA) was used to determine the molecular weight of the sample. The mobile phase consisted of 0.1 mol/L sodium chloride solution, delivered at a flow rate of 0.5 mL/min, with the column temperature maintained at 40 °C. An injection volume of 0.1 mL was applied. Prior to analysis, the sample was dissolved in the same mobile phase to a final concentration of 2 mg/mL.

#### 2.2.2. FT-IR Spectroscopy

The sample (2 mg) was ground with 100 mg of oven-dried KBr (dried at 120 °C for 24 h), and the mixture was compressed into a pellet. The infrared spectrum was then recorded using a Fourier transform infrared spectrometer (Bruker Tensor 27, Saarbrücken, Germany).

#### 2.2.3. Scanning Electron Microscopy (SEM)

The samples were evenly laid on the mica sheet and sputtered with a 20 nm thick gold film in a vacuum. Shooting was performed using SEM (Hitachi SU8010, Tokyo, Japan).

### 2.3. Animal Experiment

#### 2.3.1. Animal Model and Drug Administration

Male C57BL/6J mice, aged six to seven weeks, were obtained from Beijing Vital River Laboratory Animal Technology Co., Ltd. (Beijing, China). All animals were housed under a 12 h light/dark cycle with free access to drinking water in a temperature-controlled room (25 ± 1 °C, 55 ± 5% humidity). Following a two-week acclimatization, 30 mice were randomly divided into three groups (*n* = 10 per group) and housed separately by cage. Each group was fed one of three experimental diets for eight weeks: a standard chow diet (CT), a high-fat diet (HFD), or an HFD supplemented with 400 mg/kg body weight of SOS (HFD + SOS). The CT and HFD groups received 100 µL of distilled water by gavage daily between 9:00 and 10:00 a.m., while the HFD + SOS group received the same volume of distilled water containing 400 mg/kg SOS. The dietary compositions were as follows: CT (control diet: 19.2% protein, 4.3% fat, 67.3% carbohydrate, 4057 kcal/kg), HFD (23.7% protein, 23.6% fat, 41.4% carbohydrate, 4057 kcal/kg), and HFD + SOS (same as HFD). The complete feed composition is provided in Table S1. It should be noted that the CT diet was a fiber-free, isoenergetic purified diet rather than standard laboratory chow. This design was chosen to strictly control dietary fiber content and isolate the effects of soybean oligosaccharides, but it also represents a non-standard physiological control condition. The dietary intervention and agent administration began at the same time. Body weight and food intake were recorded weekly for each mouse. All animal procedures received ethical approval from the Institutional Animal Care and Use Committee (IACUC) of Inner Mongolia Medical University (Permit No. YKD202401207).

#### 2.3.2. Oral Glucose Tolerance Test

One week before the end of the study, mice were fasted for 6 h and then received an oral glucose challenge (2 g/kg body weight) by gavage. Blood glucose was measured at the following time points: 30 min before glucose administration (−30 min), immediately prior to gavage (0 min), and 15, 30, 60, 90, and 120 min after glucose loading. Blood samples were collected from the tail vein tip, and glucose levels were determined using a blood glucose meter (Accu-Check, Roche, Basel, Switzerland).

#### 2.3.3. Tissue Sampling and Analysis

Mice were euthanized via cervical dislocation, and tissues were immediately removed and snap-frozen in liquid nitrogen. For the collection and processing of the jejunum, ileum, cecum, and colon, 2 mm segments of the colon were cleared of luminal contents with PBS. These intestinal segments were fixed in Carnoy’s solution (ethanol:acetic acid:chloroform = 6:3:1, *v*/*v*) for 2 h at 4 °C, followed by dehydration in absolute ethanol for 24 h. Tissues were then embedded in paraffin and cut into 5-μm sections for staining. Deparaffinized sections were oxidized in 0.5% periodic acid (10 min), incubated in Schiff’s reagent (30 min, dark), and counterstained with hematoxylin. After dehydration, sections were mounted with neutral resin. For antigen retrieval, sections were heated in citrate buffer using a microwave, and endogenous peroxidase was blocked with 3% H_2_O_2_ (30 min, room temperature). Sections were then blocked with 5% BSA (1 h, RT) and incubated overnight at 4 °C with rabbit anti-MUC2 primary antibody (1:500, PA5-79702, Thermo Fisher, Waltham, MA, USA). HRP-conjugated secondary antibody was applied for 45 min at RT, followed by DAB development and hematoxylin counterstaining. Additional tissue samples were fixed in 4% paraformaldehyde (4 °C, 24 h), paraffin-embedded, and sectioned at 4 μm. These sections were stained with hematoxylin (5 min), rinsed, dipped in 1% acetic acid (5 × 30 s), washed, and counterstained with eosin. All images were captured using a light microscope (Olympus, Tokyo, Japan).

#### 2.3.4. Blood Biochemical Indexes

Commercial ELISA kits were used to quantify serum levels of interleukin-6 (IL-6), tumor necrosis factor-α (TNF-α), and lipopolysaccharide (LPS), following the manufacturers’ instructions. IL-6 and TNF-α were measured using arigoQIK^®^ ELISA Development Kits (ARG83587 and ARG83544, respectively; arigo Biolaboratories, Taiwan, China). LPS levels were determined with a Mouse LPS ELISA Kit (CSB-E13066m, Cusabio, Wuhan, China). All samples were run in duplicate, and the absorbance at 450 nm was recorded on a microplate reader. The concentrations were calculated from kit-specific standard curves.

#### 2.3.5. Measurement of Fecal TG and TC

Commercial assay kits (Applygen Technologies, Beijing, China) were used to determine fecal triglyceride (TG) and total cholesterol (TC) concentrations, following the manufacturer’s protocols.

#### 2.3.6. 16S rRNA Sequencing

Fecal total DNA was isolated using the QIAamp DNA Stool Mini Kit (Qiagen, Hilden, Germany) and preserved at −20 °C. A microspectrophotometer was employed to assess DNA concentration and purity (A260/A280). The V3–V4 hypervariable region of the 16S rDNA gene was PCR-amplified with universal primers 27F and 533R on an ABI GeneAmp^®^ 9700 thermal cycler (Applied Biosystems, Carlsbad, CA, USA). Amplicon yields were quantified using a QuantiFluor™-ST handheld fluorometer (Promega, Madison, WI, USA) and then submitted to Majorbio (Shanghai, China) for library construction and Illumina sequencing.

Raw sequencing reads underwent quality filtering, assembly, and chimera removal. High-quality sequences were then clustered into operational taxonomic units (OTUs) at a 97% similarity threshold, with a sequencing error rate below 1% and Q20 > 97%, for subsequent analyses.

#### 2.3.7. RNA Isolation and Quantitative Real-Time PCR (qRT-PCR) for Gene Expression

Owing to unexpected sample loss during experiments, only 6 valid biological replicates per group were included in the final analysis. Intestinal tissue segments were homogenized in Trizol reagent for total RNA isolation. Reverse transcription was then carried out using Prime Script reverse transcriptase and random primers to synthesize complementary DNA (cDNA) from the extracted RNA. Real-time quantitative PCR was performed on an ABI Q7 Flex system with Power SYBR Green PCR master mix (Applied Biosystems, Carlsbad, CA, USA) to assess gene expression. Relative transcript levels were calculated using the ΔΔCt method, with high-fat diet (HFD)-fed mice serving as the calibrator group. GAPDH was used as the internal reference gene. The primer sequences employed in this study are listed in Table S2.

### 2.4. Statistical Analysis

Prior to statistical analysis, the Grubbs test was used to detect outliers. All statistical analyses were performed using GraphPad Prism (version 9.5, San Diego, CA, USA). Data are expressed as mean ± standard error of the mean (s.e.m.). One-way ANOVA with Tukey’s post hoc test was applied to evaluate treatment effects, while non-parametric data were compared using the Kruskal–Wallis test followed by Dunn’s multiple comparison test. The threshold for statistical significance was *p* < 0.05. In two-way ANOVA followed by Bonferroni’s post hoc test, different subscript letters denote significant differences (*p* < 0.05).

## 3. Results

### 3.1. Structural Characterization

#### 3.1.1. Molecular Weight Analysis

GPC/MALLS analysis revealed the molecular weight of crude SOS to be 1019 Da (Table S3).

#### 3.1.2. FT-IR and SEM Analysis

**Figure 1:**
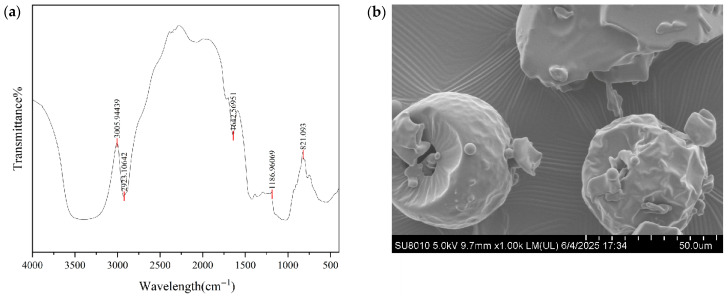
FT-IR and SEM analysis of SOS: (**a**) FT-IR spectrum; (**b**) scanning electron microscopy.

In the Fourier transform infrared spectrum, a typical saturated C-H stretching vibration peak at 2923 cm^−1^ confirmed the presence of a carbohydrate hydrocarbon skeleton (i.e., C-H bonds in monosaccharide units) within the oligosaccharide molecules. Meanwhile, the stretching vibration of glycosidic linkages in oligosaccharide molecules, observed at 1187 cm^−1^, corresponds to the core peak in the “characteristic fingerprint region” of oligosaccharides (1000~1200 cm^−1^), directly verifying the existence of the oligosaccharide backbone (Figure 1a). Scanning electron microscopy revealed that the oligosaccharide particles were morphologically heterogeneous, consisting of well-defined spherical or subspherical particles, irregularly deformed structures, and fragmented forms—features indicative of variations in molecular assembly behavior (Figure 1b). The spheroidal structure, suitable particle size, high specific surface area and abundant adhesion sites of SOS provide the core structural basis for its intestinal retention, microbiota regulation, as well as its modulatory effects on the intestinal barrier and mucus layer.

### 3.2. SOS Prevented Obesity and Improved Glucose Intolerance Caused by a High-Fat Diet

**Figure 2:**
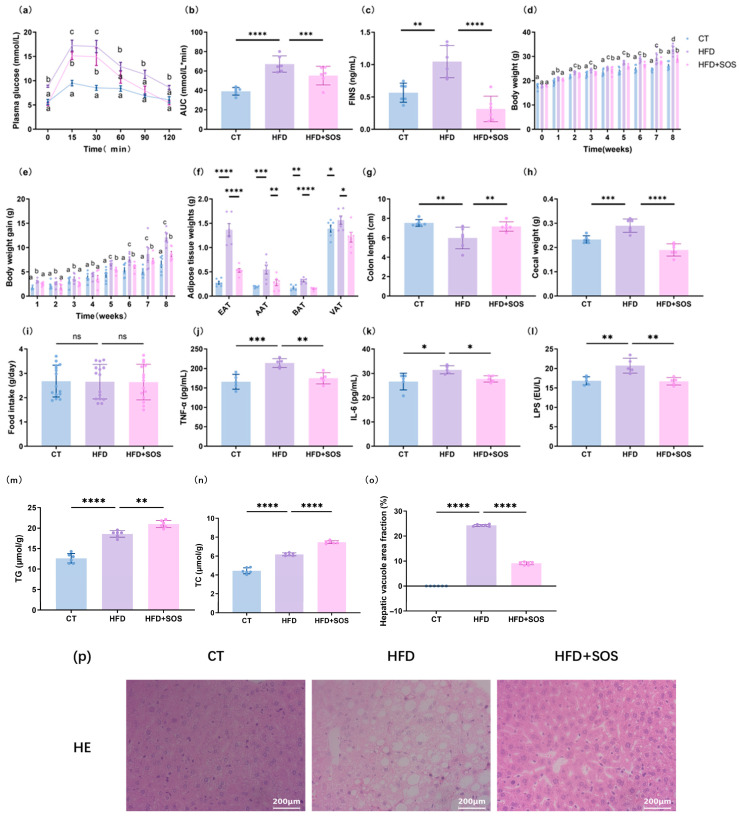
SOS supplementation can relieve obesity and glucose intolerance caused by diet (*n* = 6): (**a**) plasma glucose profile during OGTT (2 g/kg glucose oral challenge) and (**b**) corresponding area under the curve (AUC, mmol/L·min); (**c**) fasting insulin; (**d**) body weight evolution over 8 weeks (g); (**e**) net body weight gain after 8 weeks (g) (**f**) weights of epididymal (EAT), abdominal (AAT), brown (BAT), and visceral (VAT) adipose tissues (g) at 6 weeks; (**g**) colon length (cm); (**h**) cecal weight; (**i**) food intake; (**j**) serum TNF-α; (**k**) serum IL-6; (**l**) serum LPS; (**m**) fecal triglyceride; (**n**) fecal total cholesterol; (**o**) hepatic vacuole area fraction; (**p**) representative H&E-stained liver images. Treatment effects were assessed by one-way ANOVA with Tukey’s post hoc test (* *p* < 0.05, ** *p* < 0.01, *** *p* < 0.001, **** *p* < 0.0001). For panels (**a**,**d**,**e**), two-way ANOVA followed by Bonferroni’s post hoc test was applied; different subscript letters denote significant differences (*p* < 0.05).

In the oral glucose tolerance test (OGTT), SOS supplementation significantly attenuated high-fat diet-induced hyperglycemia and reduced fasting insulin levels, suggesting a potential beneficial effect of SOS on maintaining glucose homeostasis (Figure 2a–c). Notably, this metabolic improvement was positively associated with the reduced fasting insulin concentrations (Figure 2a). Compared with the HFD group, supplementing SOS led to a significant reduction in adipose tissue quality, including white adipose tissue (epididymis and visceral repository) and brown adipose tissue (Figure 2d–f). Furthermore, SOS effectively suppressed weight gain throughout the 8-week intervention period, reducing final body weight gain by 17.2% compared with the HFD group (*p* < 0.01) (Figure 2d,e). It also enhanced colon length compared to HFD groups (Figure 2g); concurrently, SOS supplementation significantly decreased cecal weight and the mass of cecal contents (Figure 2h). SOS supplementation significantly promoted fecal excretion of triglyceride (TG) and total cholesterol (TC), reduced serum levels of proinflammatory cytokines including TNF-α, IL-6 and LPS, and markedly decreased hepatic vacuole area fraction—demonstrating its protective effects against HFD-induced hepatic steatosis and systemic inflammation (Figure 2j–n). Compared with the HFD group, SOS supplementation effectively ameliorated hepatic lipid accumulation and suppressed chronic inflammatory responses, with no significant difference in food intake among all groups (Figure 2i,o,p).

### 3.3. SOS Affected Markers Involved in Intestinal Barrier Function

**Figure 3:**
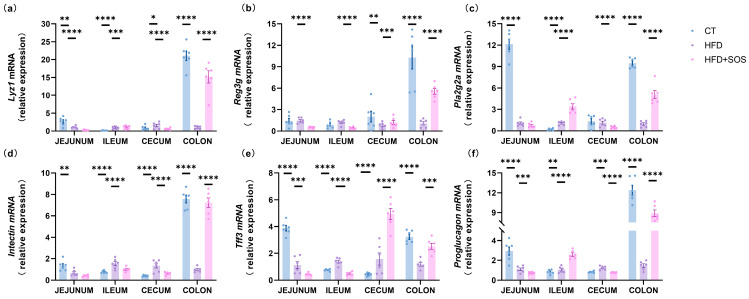
SOS promoted intestinal cell proliferation and enhanced gut barrier markers (*n* = 6). The relative mRNA expression of barrier-related genes was measured in the jejunum, ileum, cecum, and colon, including: (**a**) lysozyme C (*Lyz1*), (**b**) regenerating islet-derived 3-gamma (*Reg3g*), (**c**) phospholipase A2 group II (*Pla2g2a*), (**d**) *intectin*, (**e**) trefoil factor 3 (*Tff3*), and (**f**) *proglucagon*. One-way ANOVA with Tukey’s post hoc test was applied for parametric data, while non-parametric datasets were analyzed using the Kruskal–Wallis test followed by Dunn’s multiple comparison test. Data are presented as mean ± s.e.m. Significance levels: * *p* < 0.05, ** *p* < 0.01, *** *p* < 0.001, **** *p* < 0.0001.

Supplementation with SOS was closely associated with increased expression of intestinal barrier function markers, including antimicrobial peptides and epithelial cell turnover factors [16]. Our results demonstrated that SOS intervention significantly enhanced intestinal expression of lysozyme 1 (*Lyz1*), an antimicrobial peptide. SOS treatment also upregulated the expression of regenerating islet-derived protein 3-gamma (*Reg3g*), phospholipase A2 group IIa (*Pla2g2a*), and intelectin—an important regulator of intestinal epithelial renewal—in the colon (Figure 3a–d). Trefoil factor 3 (*Tff3*), a well-established marker of intestinal mucosal integrity and repair, was increased in the cecum and proximal colon (Figure 3e). Furthermore, SOS administration elevated colonic expression of proglucagon, a key precursor of glucagon-like peptide-1 (GLP-1) involved in glucose regulation (Figure 3f). These results suggest that SOS may contribute to improved intestinal barrier function.

### 3.4. SOS Promoted the Intestinal Mucus Production

**Figure 4:**
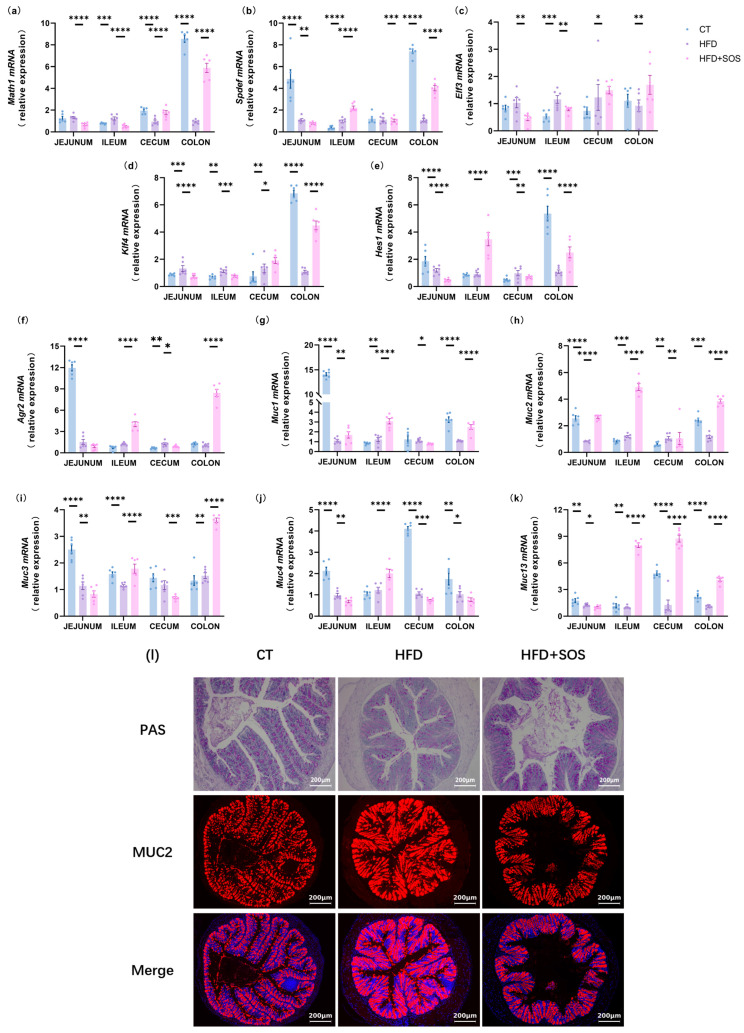
SOS enhanced goblet cell differentiation, increased goblet cell number, and upregulated markers of mucin production (*n* = 6). The relative mRNA expression of transcriptional factors involved in goblet cell differentiation was measured in the jejunum, ileum, cecum, and colon: atonal bHLH transcription factor 1 (*Math1*), SAM pointed domain containing ETS transcription factor (*Spdef*), E74-like ETS transcription factor 3 (*Elf3*), Krüppel-like factor 4 (*Klf4*), and hes family bHLH transcription factor 1 (*Hes1*) (**a**–**e**). Additionally, the expression of mucin production markers was assessed in the same intestinal segments: anterior gradient 2 (*Agr2*), mucin 1 (*Muc1*), and mucins 2, 3, 4, and 13 (*Muc2*, *Muc3*, *Muc4*, *Muc13*) (**f**–**k**). Panel (**l**) shows periodic acid-Schiff (PAS) staining and MUC2 immunofluorescence of colon sections. One-way ANOVA with Tukey’s post hoc test was applied for parametric data, while non-parametric datasets were analyzed using the Kruskal–Wallis test with Dunn’s multiple comparison test. Data are presented as mean ± s.e.m. Significance levels: * *p* < 0.05, ** *p* < 0.01, *** *p* < 0.001, **** *p* < 0.0001.

To explore the underlying mechanism, we examined whether the SOS-induced improvements in metabolism and intestinal barrier function were linked to the regulation of intestinal mucus production. Relative to the HFD group, SOS treatment markedly elevated colonic expression of progenitor-to-secretory cell differentiation markers, specifically the basic helix-loop-helix transcription factor Atoh1 (*Math1*) and Krüppel-like factor 4 (*Klf4*) (Figure 4a,d). Meanwhile, the goblet cell terminal differentiation marker *Elf3* was significantly upregulated in the colon (Figure 4c). Notably, ileal expression of Hes1, an effector of Notch signaling, was also increased (Figure 4e).

To verify whether SOS-induced increased goblet cell abundance promotes mucin secretion and subsequent formation of the protective intestinal mucus barrier, we analyzed key molecular regulatory factors. Our results demonstrated that SOS supplementation significantly upregulated colonic expression of *Muc2* (the major secreted mucin) and Agr2 (its essential post-transcriptional chaperone). Notably, these two genes were also more highly expressed in the ileum (Figure 4f,h).

Transmembrane mucins play a critical role in the mucus layer, supporting intestinal surface protection and intracellular signaling. We found that SOS supplementation significantly upregulated colonic expression of *Muc1* and *Muc3*. Additionally, *Muc13* expression was increased in the ileum, cecum, and colon (Figure 4g,i–k). Co-staining of serial crypt-to-surface sections with PAS and MUC2 immunofluorescence showed a pronounced thinning of the colonic mucus layer in HFD-fed mice—an effect that was nearly reversed by SOS supplementation (Figure 4l). After SOS intervention, the thickness of the intestinal mucus layer in mice increased significantly, indicating that the integrity of the intestinal barrier was improved; the improvement of intestinal barrier function can reduce the entry of lipopolysaccharide into the blood, thereby reducing the level of peripheral inflammation, ultimately alleviating the obesity phenotype induced by high-fat diet, forming a complete regulatory pathway of “mucus layer thickening → intestinal barrier strengthening → anti-obesity”. This finding may indicate that SOS restores the HFD-induced impaired mucus barrier by enhancing overall mucin secretion and upregulating MUC2, and collectively provides morphometric evidence supporting SOS’s role in maintaining intestinal homeostasis.

### 3.5. SOS Increased the Expression of Glycosyltransferases Related to Mucin Synthesis

**Figure 5:**
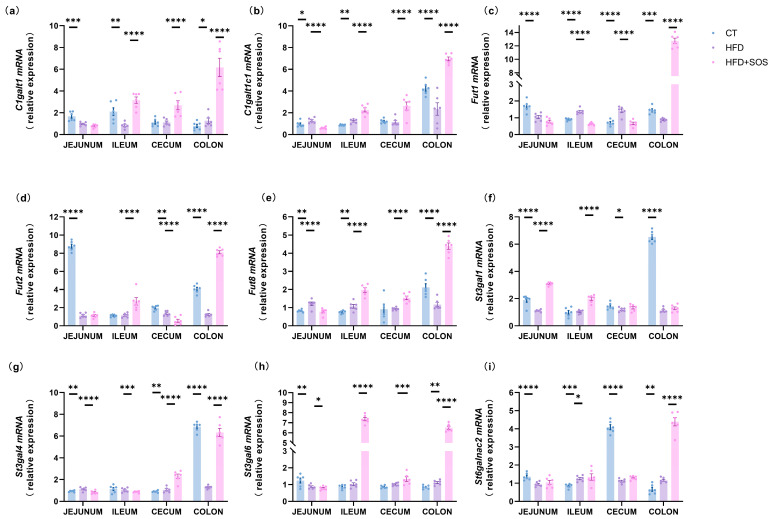
SOS increased the expression of glycosyltransferases related to mucin synthesis. Glycosyltransferase mRNA expression in jejunum, ileum, cecum, colon (*n* = 6): (**a**) *C1galt1*, (**b**) *C1galt1c1*, (**c**) *Fut1*, (**d**) *Fut2*, (**e**) *Fut8*, (**f**) *St3gal1*, (**g**) *St3gal4*, (**h**) *St3gal6*, (**i**) *St6galnac2*. Statistics: one-way ANOVA (Tukey) or Kruskal–Wallis (Dunn). Data: mean ± s.e.m. * *p* < 0.05, ** *p* < 0.01, *** *p* < 0.001, **** *p* < 0.0001.

Beyond examining goblet cell and mucin markers, we further measured the expression of critical glycosyltransferases involved in mucin modification. These enzymes participate in regulating mucin glycosylation profiles by catalyzing the site-specific addition of glycans to the protein core, a process that has been reported to influence mucus properties and function [17,18]. Of note, the expression of *C1galt1* (core 1 β1,3-galactosyltransferase) was elevated in the cecum and colon. Meanwhile, *C1galt1c1*—the essential molecular chaperone for *C1galt1*—exhibited significant upregulation in the ileum and colon (Figure 5a,b). SOS treatment also modulated the expression of glycosyltransferases related to glycan chain termination. Specifically, the expression levels of fucosyltransferases *Fut1*, *Fut2*, and *Fut8* were significantly increased in the colon, accompanied by elevated expression of the sialyltransferase *St3gal4* in the same segment (Figure 5c–e,g). Fut2-mediated fucosylation has been suggested to limit pathogenic bacterial adhesion to the mucus layer, whereas *St3gal4*-mediated sialylation may contribute to higher mucus viscosity and resistance to bacterial degradation, which could collectively support the intestinal mucus barrier [19]. These observations are in line with previous work by Paone et al., who demonstrated that prebiotic-induced changes in intestinal mucus glycosylation-related genes are associated with enhanced barrier protection against HFD-related metabolic disturbances [9]. Meanwhile, the expression of sialyltransferase, *St3gal6* and *St3gal6nac2* was elevated in both the ileum and colon (Figure 5h,i).

### 3.6. SOS Increased Markers of Mucus Secretion

**Figure 6:**
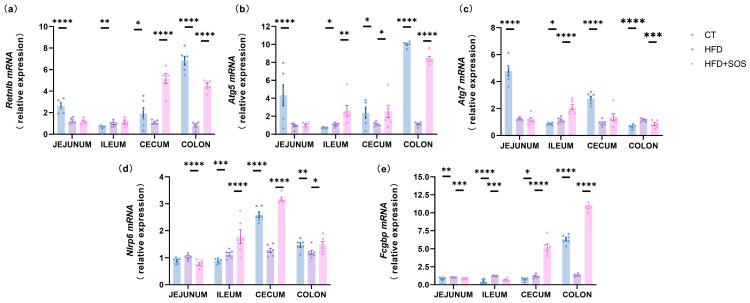
SOS upregulated markers of mucus secretion (*n* = 6). The relative mRNA expression of secretion-related genes was measured, including (**a**) resistin-like beta (*Retnlb*), (**b**,**c**) autophagy proteins 5 and 7 (*Atg5*, *Atg7*), (**d**) NOD-like receptor family pyrin domain containing 6 (*Nlrp6*), and (**e**) Fc gamma binding protein (*Fcgbp*). One-way ANOVA with Tukey’s post hoc test was used for parametric data; non-parametric datasets were analyzed using the Kruskal–Wallis test with Dunn’s multiple comparison test. Data are expressed as mean ± s.e.m. Significance: * *p* < 0.05, ** *p* < 0.01, *** *p* < 0.001, **** *p* < 0.0001.

**Figure 7:**
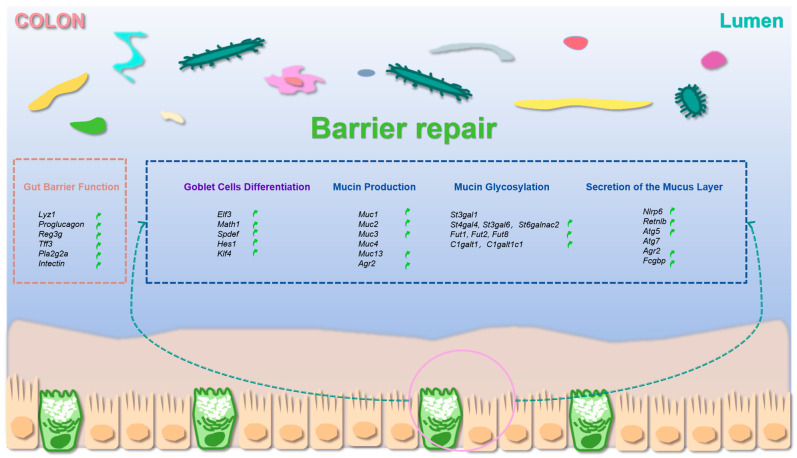
A summary figure presenting 31 biomarkers detected in the jejunum, ileum, cecum, and colon. These biomarkers are associated with intestinal barrier function, as well as the production, glycosylation, and secretion of mucins, and their levels were determined using RT-qPCR. Each biomarker is placed within a small gray box. Green arrows point to the biomarkers that showed a significant increase, specifically in the colon, which was attributed to SOS supplementation.

During the synthesis, glycosylation, and secretory vesicle packaging of MUC2 in goblet cells—key processes that ultimately lead to cellular release and the formation of the protective mucus layer—we detected a significant upregulation of the bactericidal protein resistin-like molecule β (*Retnlb*) in the cecum and colon (Figure 6a). In the colon, the expression of the autophagy-related gene *Atg5* was significantly increased (Figure 6b). Additionally, we assessed the expression of Nod-like receptor family pyrin domain containing 6 (*Nlrp6*)—a key regulator that promotes autophagy-dependent mucus secretion in goblet cells. Our results revealed an upregulation of *Nlrp6* expression in the colon (Figure 6d). In our study, *Fcgbp* expression was elevated in both the cecum and colon (Figure 6e). All the data regarding mRNA expression that are discussed in the context of the colon are illustrated in a schematic form in Figure 7. Given that the intestinal mucus layer and gut microbiota are mutually regulatory—with the mucus layer providing a protective niche for commensal microbes and microbes modulating mucus synthesis/degradation via their metabolites—we further analyzed the fecal microbiota composition to explore whether SOS-induced mucus modulation is associated with microbial shifts [9].

### 3.7. Fecal Gut Microbiota Composition Is Modulated by Dietary Treatment Protocols

The gut microbiota profoundly influences both the function and composition of intestinal mucus, and this microbial community is itself substantially modulated by dietary components [20,21]. Thus, upon completion of the treatment, we assessed the gut microbiota composition in fecal samples and quantified their relative abundances.

**Figure 8:**
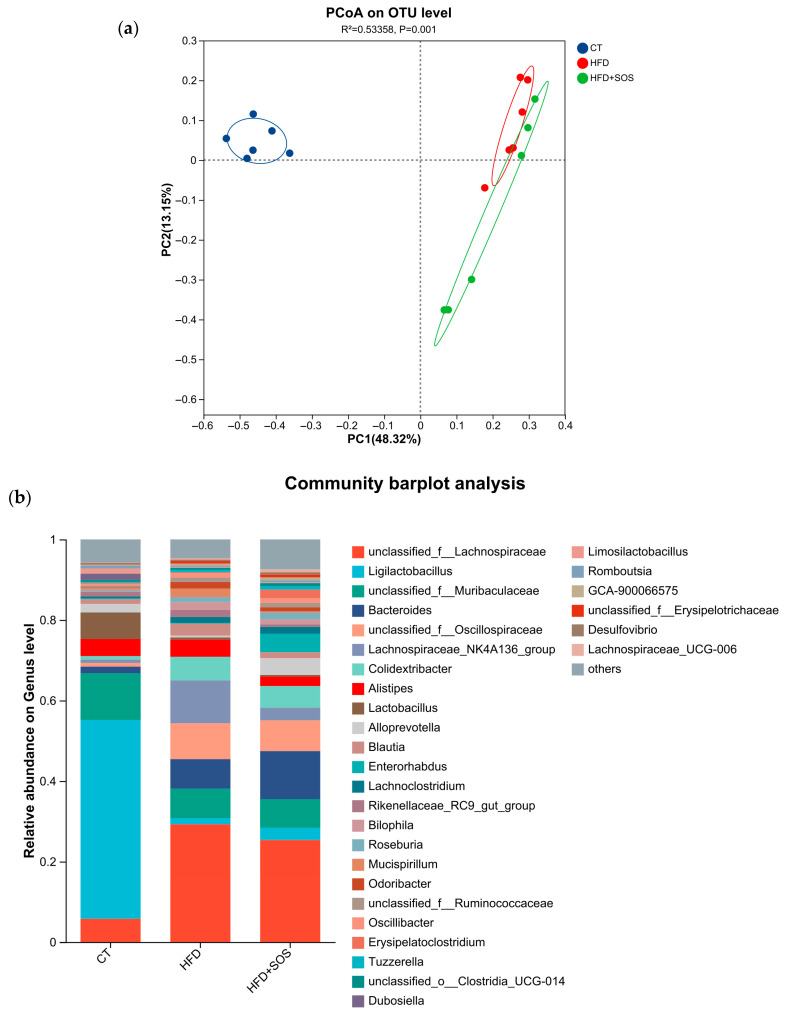
SOS induced changes in fecal gut microbiota composition (*n* = 6): (**a**) Principal coordinate analysis (PCoA) based on Bray–Curtis distances of fecal samples collected at treatment endpoint, showing microbiota clustering for the control diet (CT), high-fat diet (HFD), and HFD plus SOS (HFD + SOS) groups. (**b**) Bar plots depicting the relative abundance of gut bacteria at the genus level for each treatment group at the end of the intervention.

Principal coordinate analysis (PCoA) showed that HFD group samples clustered distinctly on the right side, indicating that the high-fat diet was linked to substantial shifts in the overall gut microbiota architecture. Although samples from the HFD + SOS group also clustered on the right, they were clearly separated from the HFD group. This observation indicates that SOS was related to a distinct microbial configuration and may be associated with a partial restoration of microbiota homeostasis disturbed by HFD feeding (Figure 8a). Relative to the CT group, the HFD group exhibited a marked decrease in *Ligilactobacillus* abundance and a pronounced increase in *unclassified_f__Lachnospiraceae*. Following SOS intervention, the HFD + SOS group showed a moderate rise in *Romboutsia*, a significant reduction in *unclassified_f__Lachnospiraceae*, but no notable restoration of *Ligilactobacillus* levels. The dominant genus in the CT group was *Ligilactobacillus*, whereas *unclassified_f__Lachnospiraceae* dominated both the HFD and HFD + SOS groups. These observations suggest that HFD was associated with marked shifts in microbial community structure, including decreased *Ligilactobacillus* and increased *unclassified_f__Lachnospiraceae*. Furthermore, the results indicate that SOS was correlated with restructured gut microbiota characterized by a modest enrichment of *Romboutsia* and reduced *unclassified_f__Lachnospiraceae* (Figure 8b).

### 3.8. The Interrelationship of Bacterial Genera with Metabolic, Gut Barrier, and Mucus Layer Variables

**Figure 9:**
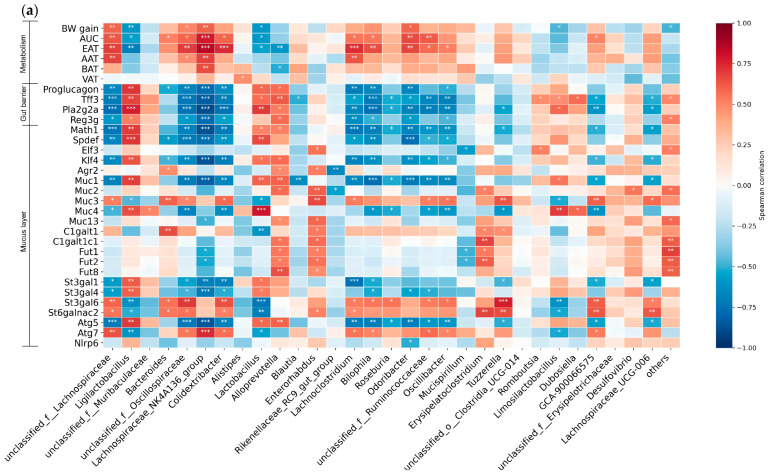

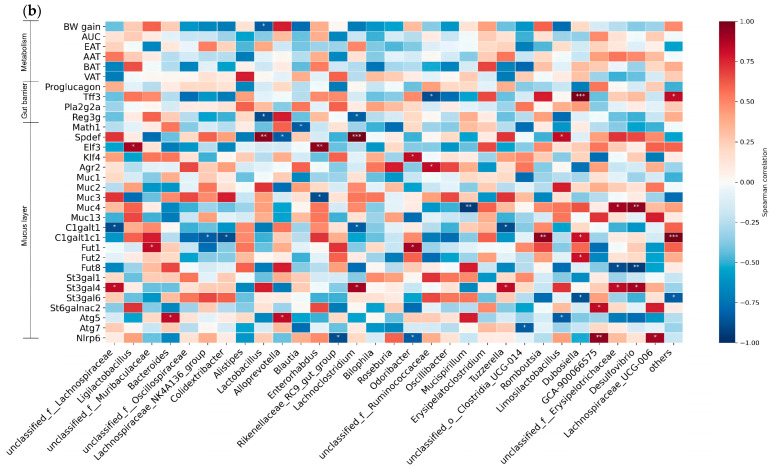
Interrelationships between bacterial genera and metabolic, gut barrier, and mucus-layer variables. (**a**,**b**) Heat maps illustrating the most significant and numerous baseline associations between gut bacterial genera (absolute abundance in feces at treatment endpoint) and parameters related to metabolism, gut barrier, goblet cells, mucin production, glycosylation, and secretion (in the colon) (*n* = 6). Panel (**a**) includes all samples from CT, HFD, and HFD + SOS groups; panel (**b**) includes only HFD + SOS samples. Statistically significant FDR-adjusted *p* values are indicated by asterisks (* *p* < 0.05, ** *p* < 0.01, *** *p* < 0.001).

We then examined the correlations between the relative abundances of specific bacterial genera and changes in metabolic parameters, intestinal barrier function, and mucus layer properties. Using a predefined list of host genes and Spearman correlation heatmap data, we analyzed strong correlations and potential functional mechanisms between key gut microbiota, host intestinal core genes, and critical metabolic indicators. Spearman correlation heatmap analysis suggested that the target gut microbiota were correlated with intestinal barrier-related genes and systemic metabolic indicators, showing distinct regulatory patterns in our dataset. *Ligilactobacillus* was significantly positively correlated with intestinal barrier repair-related gene *Tff3*, antimicrobial peptide genes (*Pla2g2a*, *Reg3g*), and epithelial differentiation-related genes (*Math1*, *Spdef*), and was significantly negatively correlated with BW gain, suggesting a potential association with the maintenance of intestinal immune barrier homeostasis and host metabolic health in the SOS intervention group. *Romboutsia* was significantly positively correlated with mucosal repair-related gene *Tff3*, epithelial differentiation-related gene *Spdef*, autophagy-related gene *Atg5*, and inflammatory regulator gene *Nlrp6*, indicating a potential link to intestinal mucosal barrier repair in the SOS intervention group. *Lachnospiraceae_UCG-006* was significantly positively correlated with glycosyltransferase-encoding genes (*C1galt1c1, St3gal1, St3gal6*), suggesting a potential association with the regulation of mucus layer glycosylation modification. *Unclassified_f__Lachnospiraceae* was significantly positively correlated with the glycosyltransferase-encoding gene *St3gal4*, while no significant correlations with other functional genes or metabolic phenotypes were detected. *Enterorhabdus* was significantly negatively correlated with the antimicrobial peptide gene *Pla2g2a*, suggesting a potential association with the regulation of intestinal innate immune function (Figure 9b).

Collectively, these findings indicate that gut microbiota is closely associated with intestinal barrier homeostasis, which may serve as a potential regulatory node linking dietary intervention and host metabolic health.

## 4. Discussion

This study systematically investigated the association between SOS supplementation and the alleviation of high-fat diet-induced obesity and related metabolic disorders. Our findings demonstrate that the beneficial phenotypes observed with SOS supplementation are associated with improved intestinal barrier function, maintained mucus layer homeostasis, and altered gut microbiota composition. These observations extend current understanding of the metabolic-related effects of SOS and provide supportive evidence for its potential use as a functional food ingredient in strategies aimed at the prevention and management of obesity and related metabolic disorders.

Specifically, SOS intervention effectively reversed the deterioration of metabolic phenotypes induced by a high-fat diet, reducing weight gain by 17.2% and improving fat accumulation and glucose tolerance. While the improvement in glucose tolerance by SOS was partly attributed to reduced body weight, our data suggest that SOS exerts a more dominant and direct role in restoring glucose homeostasis through multiple extra-weight mechanisms, including enhanced fecal lipid excretion, alleviated hepatic steatosis, reduced systemic inflammation, and upregulated proglucagon gene expression. At the intestinal barrier level, especially in the colon part, SOS upregulated the expression of multiple antimicrobial peptides (such as *Lyz1* and *Reg3g*) and epithelial renewal-related factors (such as *Tff3* and *Proglucagon*), thereby enhancing intestinal defence and repair capabilities. Consistent with previous reports that indigestible oligosaccharides are fermented by gut microbiota into short-chain fatty acids to activate intestinal L-cells and stimulate glucagon-like peptide-1 secretion for improved glucose homeostasis, our present study found that SOS treatment significantly upregulated colonic proglucagon expression, suggesting that enhanced GLP-1 synthesis may contribute to the ameliorated glucose tolerance [22]. Furthermore, it should be emphasized that indigestible oligosaccharides can be fermented by gut microbiota to produce SCFAs, which are important regulators of glucose homeostasis and lipid metabolism. Therefore, the improved glucose tolerance and reduced fat accumulation induced by SOS may also be related to the production of SCFAs. Concurrently, SOS markedly enhanced mucus barrier synthesis, modification, and secretion throughout the entire intestinal tract: by promoting expression of key transcription factors for goblet cell differentiation (e.g., *Klf4*, *Elf3*), it increased production of mucin *Muc2* and its chaperone *Agr2*; by upregulating core and terminal glycosyltransferases (e.g., *C1galt1*, *Fut2*, *St3gal4*) to optimise mucin glycosylation modifications; and by enhancing the expression of autophagy-related proteins (such as *Atg5*, *Nlrp6*) and mucin stabilising protein *Fcgbp* to promote mucus secretion and structural maintenance [23,24,25,26,27,28]. Morphological analysis further confirmed that SOS reverses high-fat diet-induced thinning of the colonic mucus layer. Notably, colonic *Muc2* expression levels showed significant negative correlations with weight gain and the area under the glucose tolerance curve, suggesting that restoration of mucus barrier function is a key component of SOS’s metabolic protective effects [29].

Gut microbiota analysis revealed that SOS intervention was associated with modulation of the gut microbial community structure altered by HFD feeding, characterized by reduced abundance of *unclassified_f__Lachnospiraceae* (the dominant genus in HFD-fed mice) and increased abundance of *Romboutsia*. Correlation analyses indicated that these key microbial shifts were associated with the expression of mucosal barrier markers and adverse metabolic parameters, suggesting that SOS may exert its effects potentially via the “microbiota–mucus” interaction axis. Notably, *Romboutsia*, enriched by SOS intervention, was significantly positively correlated with intestinal mucosal repair-related genes, consistent with previous findings that commensal microbiota associated with mucosal barrier homeostasis may contribute to the regulation of host metabolic health [30,31,32].

One limitation is the use of an isoenergetic, fiber-free control diet instead of standard chow. While this design facilitated the assessment of SOS under controlled fiber levels, it may not fully represent normal gut physiology. Moreover, although the SOS preparation contained a substantial amount of sucrose, this sugar is absorbed within the small intestine and does not reach the colon; consequently, it did not influence the primary outcomes. This constraint should be taken into consideration when interpreting the present findings. Although this study preliminarily elucidates the mechanism by which SOS exerts anti-obesity effects through a multidimensional “gut function–mucus–microbiota” network, several limitations remain. Firstly, the findings are primarily based on gene expression and microbiota composition analyses, without direct functional validation of causal relationships through experiments such as *Muc2* gene knockout or faecal microbiota transplantation. Secondly, SOS may modulate host physiology indirectly through microbial metabolites such as short-chain fatty acids, although the detailed mechanisms remain to be clarified. Since SCFAs were not measured in this study, the beneficial effects of SOS cannot be solely explained by the improvement of intestinal barrier function. The potential role of SCFAs cannot be ignored and deserves further investigation in future studies. Furthermore, this study did not systematically compare the similarities and differences in action mechanisms and functional localisation between SOS and other functional oligosaccharides (e.g., fructooligosaccharides, galactooligosaccharides). Future research may integrate multi-omics technologies, germ-free animal models, and molecular intervention approaches to comprehensively elucidate the precise pathways and key effector molecules through which SOS modulates metabolism.

In the present study, all mice were fed a fiber-free diet to avoid interference from dietary fiber on intestinal microbiota, mucus glycosylation, and intestinal barrier function. Since no fiber was supplemented in any group, the CT group only served as a control under the fiber-free condition, rather than a standard physiological control. In future studies, adding 5% cellulose to all groups would provide a more reasonable and standardized design.

## 5. Conclusions

In summary, this study systematically illustrates that SOS may alleviate high-fat diet-induced obesity and glucose metabolism disorders, possibly by restoring intestinal mucus barrier function and optimizing gut microbiota composition. These findings provide a mechanistic basis for the potential application of SOS as an anti-obesity functional ingredient, and lay a theoretical foundation for nutritional interventions targeting the gut–microbiota axis.

## Data Availability

The original contributions presented in this study are included in the article/Supplementary Material. Further inquiries can be directed to the corresponding authors.

## Supplementary Materials

The following supporting information can be downloaded at https://www.mdpi.com/article/10.3390/nu18081282/s1. Table S1: The feed ingredient list; Table S2: Reaction primers; Table S3: The molecular weight (Mw) of SOS.
