# Supplementary material for: Soybean Oligosaccharides Mitigate HFD-Induced Obesity in Mice with Changes in the Gut Mucus–Microbiota Axis
Source: Nutrients. 2026 Apr 17;18(8):1282. doi: 10.3390/nu18081282 (PMC13118999; doi:10.3390/nu18081282)
Supplement: Supplementary file 1 [file nutrients-18-01282-s001.zip › nutrients-4233201-supplementary.pdf]

Table S1. The feed ingredient list.

	CT		HFD	
**Ingredient**	**gm**	**kcal**	**gm**	**kcal**
Casein	200	800	200	800
L-Cystine	3	12	3	12
Maltodextrin 10	150	600	100	400
Sucrose	0	0	172.8	691.2
SOS	0	0	0	0
Soybean Oil	25	225	25	225
Lard	20	180	177.5	1597.5
Mineral Mix S10026	10	0	10	0
Dicalcium Phosphate	13	0	13	0
Calcium Carbonate	5.5	0	5.5	0
Potassium Citrate, 1 H_2O	16.5	0	16.5	0
Vitamin Mix V10001	10	40	10	40
Choline Bitartrate	2	0	2	0
Total	1055.1	4057	858.15	4057

Table S2. Reaction primers.

Primers	Forward Sequence	Reverse Sequence
GAPDH	CTTCTCCTGCAGCCTCGT	TCATCCACCTCCCCACAGTA
Lyz1	GCCAAGGTCTACAATCGTTGTGAGTTG	CAGTCAGCCAGCTTGACACCACG
Reg3g	CATCCACCTCTGTTGGGTTC	TTCCTGTCCTCCATGATCAAA
Pla2g2a	AAGGATCCCCCAAGGATGCCAC	CAGCCGTTTCTGACAGGAGTTCTGG
Intectin	GCACTATTGCAGAGGTCCGT	GTTGCCCCTGATTCTGCTGG
Tff3	CCCTGGTGCTTCAAACCTCT	GGGATGCTTGCTACCCTTG
Proglucagon	TGGCAGCACGCCCTTC	GCGCTTCTGTCTGGGA
Math1	CAAGTGTGTCCAGCAGTGTG	TTGAGTTTCTTCAAGGCGGC
Spdef	AGGTGCAATCGATGGTTGTG	AGGGTCTGCTGTGATGTTCA
Elf3	CCTATGAGAAGCTGAGCCGA	ACCTCTTCTTCCTTCCAGCC
Klf4	GTGCCCCGACTAACCGTTG	GTCGTTGAACTCCTCGGTCT
Hes1	CCGGCATTCCAAGCTAGAGA	GGTATTTCCCCAACACGCTC
Agr2	GCCAAAGACACCACAGTCAA	CCATCAAGGGTCTGTTGCTT
Muc1	GACATCTTTCCAACCCAGGACA	AAGAGAGACTGCTACTGCCATTAC
Muc2	ATGCCCACCTCCTCAAAGAC	GTAGTTTCCGTTGGAACAGTGAA
Muc3	CCGACACATTGCTGCTGAGAAT	GCTGTCGTCTTGGGTGCTATTT
Muc4	CTGTGTCTGAGCTGCCTGTATT	GGGTGTCTGTGTTGATGTTGTTG
Muc13	CCCTCATCCTCATCTTGCTGATT	CTCTGCTCTTCTCCATCCTTCTTT
C1galt1	ATGGACACAGTCACCTCAAAGG	GAGGTTCTCAGCAACGTCTATGT
C1galt1c1	TCTCACGTCCAAGCCTCGT	TGTGGCCTAGCATAGTGATCAAG
Fut1	AGAATTCGCTTGCACCACCA	AAGAAGGAGCCGGCAGAGA
Fut2	TGAACTTTCGGCTAAGGTACATCT	GGAAGTGGGCCAGAGGAAAG
Fut8	AGGCGAATGGCTGAGTCTCT	TGGCCTTAACAAGCTGTTCTTCT
St3gal1	GCCCACTATGCCAGACACTT	TCAGCAGAGTCAAACCCAGC
St3gal4	GGCTCTGGTCCTTGTTGTTG	TCCCTAGAACGGTTGCCAAAA
St3gal6	CACCCCAAAAGCGCAGATTTATT	CCTGCCTGAAACAGAGTCCAA
St6galnac2	CGGATGTTGTTGCTCGTTGC	AGTCGGCTCTTTCTGTTTTCC
Retnlb	CAAGGAAGCTCTCAGTCGTCAA	CACTAGTGCAGGAGATCGTCTTAG
Atg5	ATGGTTTGAATATGAAGGCACACC	TGATGTTCCAAGGAAGAGCTGAA
Atg7	CTTCCTGAGAGCATCCCTCTAATC	CGGCTCGACACAGATCATCATAG
Nlrp6	CCCGAAATGTCATCTGAGTGTTCT	TTCAGGGCCTCGGAAAGGT
Fcgbp	AACTTTGCCCACTGACCTG	CCACAGCCTCCCTGCACT

Table S3. The molecular weight (Mw) of SOS.

Parameters	Units
Molar mass moments (g/mol)	
Weight-average molecular weight (Mw)	1.019e+3(7%)
Number-average molecular weight (Mn)	8.520e+2(7%)
Peak-position molecular weight (Mp)	7.965e+2(4%)
Z-average molecular weight (Mz)	2.751e+3(25%)
Polydispersity	
Mw/Mn	1.196(10%)
